# Supplementary material for: Troponin elevation pattern and subsequent cardiac and non-cardiac outcomes: Implementing the Fourth Universal Definition of Myocardial Infarction and high-sensitivity troponin at a population level
Source: PLoS One. 2021 Mar 12;16(3):e0248289. doi: 10.1371/journal.pone.0248289 (PMC7954292; doi:10.1371/journal.pone.0248289)
Supplement: S1 Fig — Estimated hazard ratio of all-cause mortality, subsequent myocardial infarction, heart failure admission and ventricular arrhythmia or cardiac arrest in patients with A) acute myocardial infarction, B) acute myocardial injury with recognized coronary artery disease, C) acute myocardial injury without recognized coronary artery disease and D) chronic myocardial injury, relative to patients with no myocardial injury. Graphs were adjusted for age in years, sex, lowest in-hospital estimated glomerular filtration rate, maximal in-hospital high-sensitivity troponin-T, clinical comorbidities including diabetes mellitus, chronic obstructive pulmonary disease, dementia, peripheral artery disease, and previous stroke. (DOCX) [file pone.0248289.s001.docx]

**S1 Fig. Estimated hazard ratio of all-cause mortality, subsequent myocardial infarction, heart failure admission and ventricular arrhythmia or cardiac arrest in patients with A) acute myocardial infarction, B) acute myocardial injury with recognized coronary artery disease, C) acute myocardial injury without recognized coronary artery disease and D) chronic myocardial injury, relative to patients with no myocardial injury.** Graphs were adjusted for age in years, sex, lowest in-hospital estimated glomerular filtration rate, maximal in-hospital high-sensitivity troponin-T, clinical comorbidities including diabetes mellitus, chronic obstructive pulmonary disease, dementia, peripheral artery disease, and previous stroke.
