# Supplementary material for: Troponin elevation pattern and subsequent cardiac and non-cardiac outcomes: Implementing the Fourth Universal Definition of Myocardial Infarction and high-sensitivity troponin at a population level
Source: PLoS One. 2021 Mar 12;16(3):e0248289. doi: 10.1371/journal.pone.0248289 (PMC7954292; doi:10.1371/journal.pone.0248289)
Supplement: S2 Fig — Estimated hazard ratio of pneumonia (left) and neck of femur fracture (right) in patients with acute myocardial infarction, acute myocardial injury with recognized coronary artery disease, acute myocardial injury without recognized coronary artery disease and chronic myocardial injury, relative to patients with no myocardial injury. Graphs were adjusted for all variables included in the flexible parametric model. (DOCX) [file pone.0248289.s002.docx]

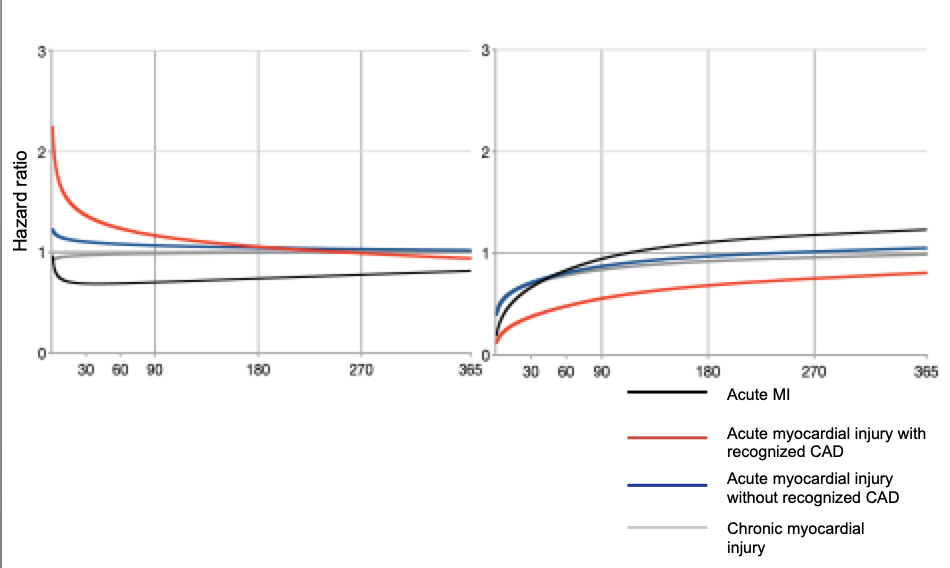


**S2 Fig. Estimated hazard ratio of pneumonia (left) and neck of femur fracture (right) in patients with acute myocardial infarction, acute myocardial injury with recognized coronary artery disease, acute myocardial injury without recognized coronary artery disease and chronic myocardial injury, relative to patients with no myocardial injury.** Graphs were adjusted for all variables included in the flexible parametric model.
