# Supplementary material for: Troponin elevation pattern and subsequent cardiac and non-cardiac outcomes: Implementing the Fourth Universal Definition of Myocardial Infarction and high-sensitivity troponin at a population level
Source: PLoS One. 2021 Mar 12;16(3):e0248289. doi: 10.1371/journal.pone.0248289 (PMC7954292; doi:10.1371/journal.pone.0248289)
Supplement: S1 Table — CAD = coronary artery disease, NOF = neck of femur. (DOCX) [file pone.0248289.s003.docx]

**S1 Table. Observed incidence of pneumonia and neck of femur fracture at 1-year.** CAD**=**coronary artery disease**,** NOF=neck of femur.

| **N = 372,310** | **Acute myocardial infarction**  **(n=19,052)** | **Acute injury with recognized CAD**  **(n=6,928)** | **Acute injury without recognized CAD**  **(n=32,231)** | **Chronic myocardial injury**  **(n=55,056)** | **No myocardial injury**  **(n=259,043)** |
| --- | --- | --- | --- | --- | --- |
| **1-year Pneumonia (%)** | 4  (0.02) | 4  (0.06) | 58  (0.18) | 107  (0.19) | 416  (0.16) |
| **1-year NOF fracture (%)** | 2  (0.01) | 4  (0.06) | 7  (0.02) | 13  (0.02) | 27  (0.01) |
