# Supplementary material for: Troponin elevation pattern and subsequent cardiac and non-cardiac outcomes: Implementing the Fourth Universal Definition of Myocardial Infarction and high-sensitivity troponin at a population level
Source: PLoS One. 2021 Mar 12;16(3):e0248289. doi: 10.1371/journal.pone.0248289 (PMC7954292; doi:10.1371/journal.pone.0248289)
Supplement: S2 Table — CAD = coronary artery disease. (DOCX) [file pone.0248289.s004.docx]

**S2 Table. Diagnoses based on troponin pattern and diagnostic code versus adjudicated diagnoses.** CAD=coronary artery disease.

| **Adjudicated diagnosis (below)** | **Acute myocardial infarction**  **(n=323)** | **Acute myocardial injury with recognized CAD**  **(n=284)** | **Acute myocardial injury without recognized CAD**  **(n=765)** | **Chronic myocardial injury**  **(n=887)** | **No myocardial injury**  **(n=4103)** |
| --- | --- | --- | --- | --- | --- |
| **Acute myocardial infarction** | 240 | 40 | 17 | 8 | 4 |
| **Acute myocardial injury with recognized CAD** | 11 | 108 | 86 | 12 | 0 |
| **Acute myocardial injury without recognized CAD** | 35 | 109 | 407 | 29 | 22 |
| **Chronic myocardial injury** | 35 | 27 | 246 | 830 | 224 |
| **No myocardial injury** | 2 | 0 | 9 | 8 | 3853 |
