# Supplementary material for: Troponin elevation pattern and subsequent cardiac and non-cardiac outcomes: Implementing the Fourth Universal Definition of Myocardial Infarction and high-sensitivity troponin at a population level
Source: PLoS One. 2021 Mar 12;16(3):e0248289. doi: 10.1371/journal.pone.0248289 (PMC7954292; doi:10.1371/journal.pone.0248289)
Supplement: S3 Table — The model adjusted for age in years, sex, lowest in-hospital estimated glomerular filtration rate, maximal in-hospital high-sensitivity troponin-T, clinical comorbidities such as diabetes mellitus, chronic obstructive pulmonary disease, dementia, peripheral artery disease, and previous stroke. CAD = coronary artery disease, HR = hazard ratio, 95%CI = 95% confidence interval, MI = myocardial infarction, HF = heart failure. (DOCX) [file pone.0248289.s005.docx]

**S3 Table. Estimated hazard ratios of 30-day and 1-year all-cause mortality, recurrent myocardial infarction, and subsequent heart failure admission in the acute myocardial infarction, acute myocardial injury with recognized coronary artery disease, acute myocardial injury without recognized coronary artery disease and chronic myocardial injury groups** **after excluding patients with 4 or more non-cardiovascular presentation per year.**  The model adjusted for age in years, sex, lowest in-hospital estimated glomerular filtration rate, maximal in-hospital high-sensitivity troponin-T, clinical comorbidities such as diabetes mellitus, chronic obstructive pulmonary disease, dementia, peripheral artery disease, and previous stroke. CAD=coronary artery disease, HR=hazard ratio, 95%CI=95% confidence interval, MI=myocardial infarction, HF=heart failure.

|  | **All-cause mortality** | **Recurrent MI** | **HF admission** |  |
| --- | --- | --- | --- | --- |
| **Acute myocardial infarction (n=17410)** | | | | |
| **30-day HR (95%CI)** | 1.42 (1.00-2.02) | 8.88 (7.83-10.08) | 2.05 (1.80-2.32) |  |
| **1-year HR (95%CI)** | 1.31 (1.14-1.50) | 5.99 (5.5-6.52) | 1.02 (0.93-1.12) |  |
| **Acute myocardial injury with recognized CAD (n=5980)** | | | | |
| **30-day HR (95%CI)** | 4.08 (2.90-5.74) | 3.22 (2.49-4.16) | 2.21 (1.87-2.60) |  |
| **1-year HR (95%CI)** | 2.31 (2.02-2.66) | 2.68 (2.32-3.10) | 1.29 (1.17-1.43) |  |
| **Acute myocardial injury without recognized CAD (n=26374)** | | | | |
| **30-day HR (95%CI)** | 5.74 (2.71-2.05) | 2.23 (1.84-2.72) | 1.82 (1.64-2.02) |  |
| **1-year HR (95%CI)** | 1.94 (1.76-2.14) | 1.63 (1.44-1.83) | 1.30 (1.21-1.40) |  |
| **Chronic myocardial injury (n=41119)** | | | | |
| **30-day HR (95%CI)** | 3.11 (1.72- 3.59) | 2.28 (1.96-2.65) | 1.83 (1.68-2.00) |  |
| **1-year HR (95%CI)** | 1.49 (1.37-1.61) | 2.13 (1.96-2.32) | 1.55 (1.49-1.63) |  |
