# Supplementary material for: Troponin elevation pattern and subsequent cardiac and non-cardiac outcomes: Implementing the Fourth Universal Definition of Myocardial Infarction and high-sensitivity troponin at a population level
Source: PLoS One. 2021 Mar 12;16(3):e0248289. doi: 10.1371/journal.pone.0248289 (PMC7954292; doi:10.1371/journal.pone.0248289)
Supplement: S4 Table — Cox regression models adjusted for age in years, sex, lowest in-hospital estimated glomerular filtration rate, maximal in-hospital high-sensitivity troponin-T, clinical comorbidities including diabetes mellitus, chronic obstructive pulmonary disease, dementia, peripheral artery disease, and previous stroke. CAD = coronary artery disease, HR = hazard ratio. *This group includes both the acute myocardial infarction and the acute myocardial injury with recognized coronary artery disease groups. (DOCX) [file pone.0248289.s006.docx]

**S4 Table. Adjusted hazard ratios of new/recurrent myocardial infarction and subsequent heart failure admission in patients with acute myocardial injury with recognized coronary artery disease, acute myocardial injury without recognized coronary artery disease and chronic myocardial injury based on multivariable Cox regression models.** Cox regression models adjusted for age in years, sex, lowest in-hospital estimated glomerular filtration rate, maximal in-hospital high-sensitivity troponin-T, clinical comorbidities including diabetes mellitus, chronic obstructive pulmonary disease, dementia, peripheral artery disease, and previous stroke. CAD=coronary artery disease, HR=hazard ratio. *This group includes both the acute myocardial infarction and the acute myocardial injury with recognized coronary artery disease groups.

| **Prior event frequency** | **One prior event** | **Two prior events** | **≥ Three prior events** |
| --- | --- | --- | --- |
| **Adjusted HR for subsequent myocardial infarction** | | | |
| Acute myocardial injury with CAD* | 2.39 (2.12-2.70) | 5.65 (4.55-7.02) | 12.09 (8.98-16.26) |
| Acute myocardial injury without CAD | 1.89 (1.59-2.26) | 2.50 (1.52-4.11) | 2.18 (0.70-6.79) |
| Chronic myocardial injury | 1.74 (1.50-2.01) | 2.08 (1.54-2.81) | 3.30 (2.32-4.70) |
| **Adjusted HR for subsequent heart failure admission** | | | |
| Acute myocardial injury with CAD* | 1.67 (1.53-1.85) | 2.39 (1.95-2.95) | 3.82 (2.71-5.39) |
| Acute myocardial injury without CAD | 2.19 (2.02-2.37) | 3.28 (2.74-3.92) | 4.30 (2.94-6.30) |
| Chronic myocardial injury | 1.96 (1.83-2.09) | 3.41 (3.08-3.77) | 4.77 (4.22-5.39) |
